# 572. Real-world Effectiveness of COVID-19 mRNA Vaccines against Hospitalizations and Deaths in a Retrospective Cohort

**DOI:** 10.1093/ofid/ofab466.770

**Published:** 2021-12-04

**Authors:** Farhaan S Vahidy, Lauren Pischel, Mauricio E Tano, Alan Pan, Marc L Boom, Henry Sostman, Khurram Nasir, Saad Omer

**Affiliations:** 1 Houston Methodist, Houston, Texas; 2 Yale School of Medicine, New Haven, California; 3 Houston Methodist Hospital, Houston, Texas; 4 Yale Institute for Global Health

## Abstract

**Background:**

The effectiveness of Severe Acute Respiratory Syndrome Coronavirus 2 vaccines after two doses needs to be demonstrated beyond clinical trials.

**Methods:**

In a retrospective cohort assembled from a cross-institution comprehensive data repository, established patients of the health care system were categorized as having received no doses, one dose or two doses of SARS-CoV-2 mRNA vaccine through April 4, 2021. Outcomes were COVID-19 related hospitalization and death.

**Results:**

Of 94,018 patients 27.7% had completed two doses and 3.1% had completed one dose of a COVID-19 mRNA vaccine. The two dose group was older with more comorbidities. 1.0% of the two dose group had a COVID-19 hospitalization, compared to 4.0% and 2.7% in the one dose and no dose groups respectively. The adjusted Cox proportional-hazards model based vaccine effectiveness after two doses (vs. no dose) was 96%(95% confidence interval(CI):95–97), compared to 78%(95%CI:76–82) after one dose. After two doses, vaccine effectiveness for COVID-19 mortality was 97.9%(95%CI:91.7–99.5), and 53.5%(95%CI:0.28–80.8) after one dose. Vaccine effectiveness at preventing hospitalization was conserved across age, race, ethnicity, Area Deprivation Index and Charlson Comorbidity Indices.

Cohort Enrollment and Distribution by Immunization Status and Vaccine effectiveness against mortality

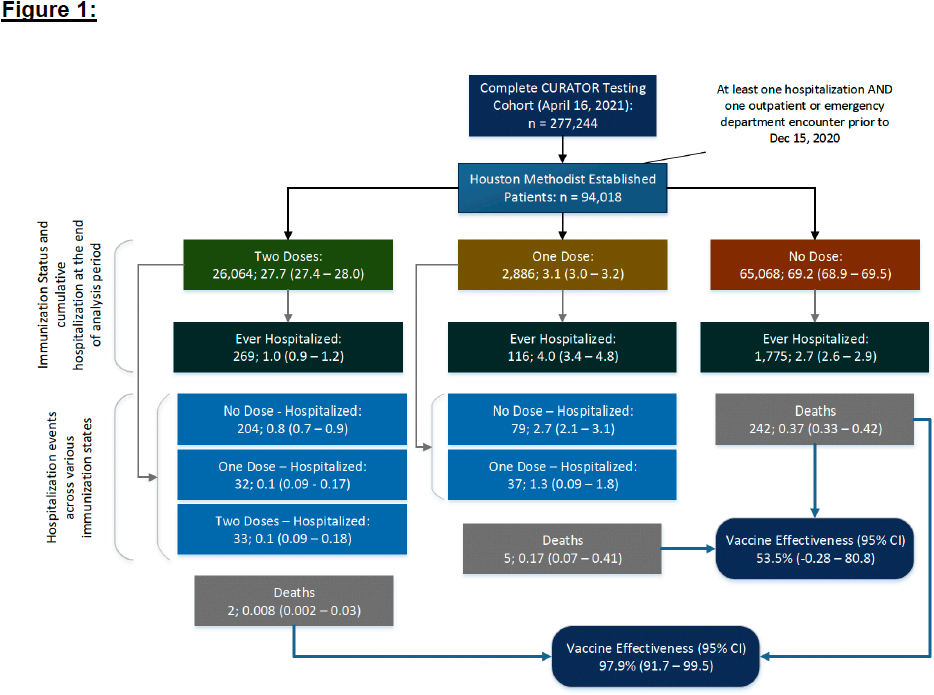

Cohort members are described by their immunization status and hospitalization at the end of the study period ending April 4th, 2021. Percentages compare this population to the total established patients. Each group is then divided into when hospitalized events occurred across immunization status. These percentages compare the number of events to the population in the immunization status at the end of the analysis period. Odds ratios for mortality were calculated and vaccine effectiveness calculated as 1 minus odds ratio times 100%.

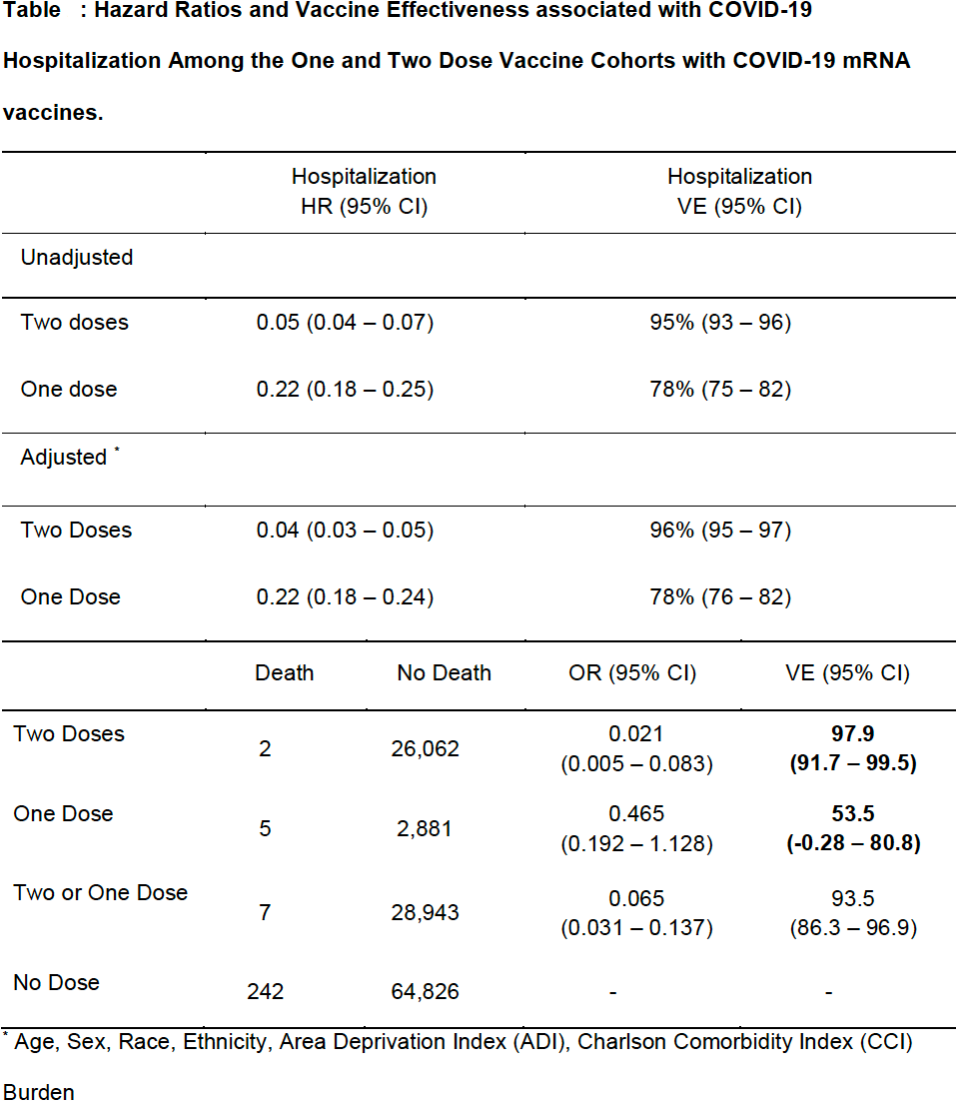

**Conclusion:**

In a large, diverse US cohort, receipt of two doses of an mRNA vaccine was highly effective in the real-world at preventing COVID-19 related hospitalizations and deaths with a substantive difference in effectiveness between one and two doses.

**Disclosures:**

**All Authors**: No reported disclosures

